# Uncertainty Analysis of Knowledge Reductions in Rough Sets

**DOI:** 10.1155/2014/576409

**Published:** 2014-08-27

**Authors:** Ying Wang, Nan Zhang

**Affiliations:** ^1^Department of Computer Science and Technology, Tongji University, 4800 Caoan Road, Shanghai 201804, China; ^2^Department of Computer and Control Engineering, Yantai University, 32 Qingquan Road, Shandong 264005, China

## Abstract

Uncertainty analysis is a vital issue in intelligent information processing, especially in the age of big data. Rough set theory has attracted much attention to this field since it was proposed. Relative reduction is an important problem of rough set theory. Different relative reductions have been investigated for preserving some specific classification abilities in various applications. This paper examines the uncertainty analysis of five different relative reductions in four aspects, that is, reducts' relationship, boundary region granularity, rules variance, and uncertainty measure according to a constructed decision table.

## 1. Introduction

Uncertainty is associated with randomness, fuzziness, vagueness, roughness, and incomplete knowledge. The theories of probability, information, fuzzy set, evidence set, rough set, and so forth have been used for uncertainty analysis [1]. Rough set theory (RST) as a new comer and one of methods for the representation of uncertainty has gained an increasing attention from both the theoretical and the applied points of view. Uncertainty exists in realistic world inherently. There are many factors affecting the uncertainty of actual questions. Examining and analyzing the characteristics of uncertainty from various situations are vital in intelligent information processing.

Attribute reduction is an important problem of rough set theory. A reduct is a minimum subset of attributes that provides the same description or classification ability as the entire set of attributes [2]. A relative reduct in Pawlak rough set is a minimum subset of attributes that preserves the positive region of the classification unchangeable, and it can also be defined as a minimum subset of condition attributes that provides the same classification ability as the entire set of attributes in a decision table. Many objective functions for attribute reduction have been proposed and examined to find the reduct to preserve a specific property [3–10]. The relationship among various reductions has attracted interest of some researchers [3, 11–15]. This also inspires our interest to investigate the uncertainty variation under a reduction and the uncertainties in various relative reducts.

There are two types of uncertainty inherently in RST. The first type of uncertainty arises from the indiscernibility relation. It increases as the granularity of the partition becomes coarser. The second one results from the approximation regions of rough sets, since the lower approximation is the certain region and the upper approximation is the possible region. This gives rise to a direction of analyzing uncertainty in relative reductions.

In this paper, the uncertainty analysis of five relative reductions is investigated in four aspects. Firstly, the relationships among different reducts are listed according to some research results. Secondly, the boundary areas of five reductions are described in detail according to a constructed decision table. Thirdly, the quality analysis and variant process of classification rules generated from these five relative reductions are discussed. Lastly, the definition of uncertainty measure in classification is proposed.

In the remainder of this paper, some related notations are reviewed and uncertainty analysis of five relative reductions is discussed.

## 2. Basic Notions

Information table is used by Pawlak for raw data representation [2]. For classification tasks, we consider a special information table with a set of decision attributes. Such an information table is also called a decision table. In this part, only some related notations are reviewed.

**Definition 1 .**

A decision table is given as the following tuple:

(1) $$\begin{array}{c} S=\left(U,A=C\cup D,\left\{V_{a}\;|\;a\in A\right\},\left\{I_{a}\;|\;a\in A\right\}\right), \end{array}$$

where *U* is a finite nonempty set of objects, *A* is a finite nonempty set of attributes including a set *C* of condition attributes that describe the objects and a set *D* of decision attributes that indicate the classes of objects, *V*_*a*_ is a nonempty set of values of *a* ∈ *A*, and *I*_*a*_ : *U* → *V*_*a*_ is an information function that maps an object in *U* to exactly one value in *V*_*a*_.

For simplicity, we assume *D* = {*d*} in this paper, where *d* is a decision attribute which labels the decision for each object. A table with multiple decision attributes can be easily transformed into a table with a single decision attribute by considering the Cartesian product of the original decision attributes.

A partition *π*_*D*_: *U*/*D* = {*D*_1_, *D*_2_,…, *D*_|*U*/*D*|_} is used to denote the partition of the universe *U* defined by the decision attribute set *D*, and so is another partition *π*_*B*_ defined by a condition attribute set *B*⊆*C*. The equivalence classes induced by the partition *π*_*B*_ are the basic blocks to construct the Pawlak rough set approximations. For a decision class *D*_*i*_ ∈ *π*_*D*_, the lower and upper approximations of *D*_*i*_ with respect to a partition *π*_*B*_ are defined by Pawlak [2]:

(2) $$\begin{array}{c} {\underline{apr}}_{\pi_{B}}\left(D_{i}\right)=\left\{x\in U\;|\;{\left[x\right]}_{B}\subseteq D_{i}\right\}\\ {\bar{apr}}_{\pi_{B}}\left(D_{i}\right)=\left\{x\in U\;|\;{\left[x\right]}_{B}\cap D_{i}\neq \emptyset \right\}. \end{array}$$

Set $BND_{\pi_{B}}(D_{i})={\bar{apr}}_{\pi_{B}}(D_{i})-{\underline{apr}}_{\pi_{B}}(D_{i})$ will be called the *π*_*B*_-boundary of *D*_*i*_.

**Definition 2 .**

The positive and boundary regions of the partition *π*_*D*_ with respect to a partition *π*_*B*_ are defined by Yao [16]:

(3) $$\begin{array}{c} {\text{POS}}_{\pi_{B}}\left(\pi_{D}\right)={⋃}_{1\leq i\leq m}{\text{POS}}_{\pi_{B}}\left(D_{i}\right);\\ {\text{BND}}_{\pi_{B}}\left(\pi_{D}\right)={⋃}_{1\leq i\leq m}{\text{BND}}_{\pi_{B}}\left(D_{i}\right). \end{array}$$

**Theorem 3 .**

In a decision table *S*, the positive and boundary regions of the partition *π*_*D*_ with respect to a partition *π*_*B*_ have the following properties [16].

1. If the decision table is consistent, then *POS*_*π*_*B*__(*π*_*D*_) = *U* and *BND*_*π*_*B*__(*π*_*D*_) = *∅*.
2. If the decision table is inconsistent, then *POS*_*π*_*B*__(*π*_*D*_) ∪ *BND*_*π*_*B*__(*π*_*D*_) = *U* and *BND*_*π*_*B*__(*π*_*D*_) ≠ *∅*.

**Proposition 4 .**

In a decision table *S*, if *BND*_*π*_*C*__(*π*_*D*_) ≠ *∅*, then S is inconsistent; otherwise, it is consistent.

Decision rule generation is an important issue in a decision table. The rough set approach offers all solutions to the problem of decision table simplification and many real applications have been found in various fields [17]. Rule sets may include certain and uncertain (or possible) rules.  Theorem 3 can be written as Theorem 5 in the view of decision rules.

**Theorem 5 .**

In a decision table *S*, the rule sets have the following properties.

1. If the decision table is consistent, then any rule of the rule sets is a certain rule.
2. If the decision table is inconsistent, then the rule sets consist of certain rules and uncertain rules. And the number of uncertain rules is larger than zero.

**Definition 6 .**

In a decision table *S*, confidence of uncertainty rules of *S* with respect to a condition attribute set *B*⊆*C* and an equivalence class *B*_*j*_ ⊂ *π*_*B*_ is defined as

(4) κ(Di ∣ Bj)=|(x,y)∈Bj ∣ fd(x)=fd(y)=Di||(x,y)∈Bj|,i=1,2,…,|UD ∣ Bj|,

where 0 ≤ *κ* ≤ 1 obviously. *κ*(*D*_*i*_∣*B*_*j*_) is written as *κ*_*i*_ when *B*_*j*_ is known. All the rules with respect to *B*_*j*_ in different *D*_*i*_ are called one pair of rules. The confidence of one pair of rules has the property that all the sum of *κ*_*i*_ is equal to 1. But the value range of *i* may be different with different subset *B*_*j*_ in partition *π*_*B*_. A rule with premises (preconditions) *P*, conclusions (post conditions) *Q*, and confidence *κ*_*i*_ is denoted as *P* → *κ*_*i*_ *Q*. If *κ*_*i*_ = 1, then the rule is certain; if *κ*_*i*_ = 0, then the rule is impossible; otherwise, it is possible or uncertain.

In order to express the degree of completeness and incompleteness of knowledge *R* about the nonempty set *X*, a pair of measures is defined by Pawlak [18]. They are the accuracy measure of $X\alpha_{R}(X)=\left(\text{card}\;\underline{R}(X)/\text{card}\;\bar{R}(X)\right)$ and *R*-roughness of $\rho_{R}(X)=1-\alpha_{R}(X)=1-\left(\text{card}\;\underline{R}(X)/\text{card}\;\bar{R}(X)\right)=\left(\text{card}\;R_{BN}(X)/\text{card}\;\bar{R}(X)\right)$.

Pawlak also defines a measure to evaluate the quality of approximation of *π*_*D*_ by *π*_*B*_ [18], *γ*_*π*_*B*__(*π*_*D*_) = (|POS_*π*_*B*__(*π*_*D*_)|/|*U*|). Similarly, *ρ*_*π*_*B*__(*π*_*D*_) = 1 − *γ*_*π*_*B*__(*π*_*D*_) = (|BND_*π*_*B*__(*π*_*D*_)|/|*U*|); a variety of *γ*_*π*_*B*__(*π*_*D*_) can be defined as and referred to as *π*_*B*_-roughness of classification or approximation. It can express the degree of inexactness of knowledge *π*_*B*_ about the classification *π*_*D*_.

This method to measure uncertainty is only associated with object numbers in the boundary region and the universe of discourse not the granularity of discourse. A modified measure, the rough entropy, is proposed by Beaubouef et al. [19], which combined the roughness of a set with approximate granularity:

(5) $$\begin{array}{c} Er\left(X\right)=-\left(\rho_{R}\left(X\right)\right)\left[\sum Q_{i}\log\left(P_{i}\right)\right]. \end{array}$$

According to Beaubouef, the rough entropy of each decision class is the probabilities for each equivalence class belonging either wholly or in part to it. There is no ordering associated with individual class members. Therefore the probability of any one value of the class being named is the reciprocal of the number of elements in the class. If *c*_*i*_ is the cardinality of, or the number of elements in, equivalence class *i* and all members of a given equivalence class are equal, then *P*_*i*_ = 1/*c*_*i*_ represents the probability of one of the values in class *i*. *Q*_*i*_ denotes the probability of equivalence class *i* within the universe. *Q*_*i*_ is computed by taking the number of elements in class *i* and dividing by the total number of elements in all equivalence classes combined.

The rough entropy *Er*(*X*) indicates the uncertain percentage concerning granularity, but it is only defined for a set. By generalizing it to a partition, a definition of rough entropy for a classification is proposed in Section 3.4.

In order to support our discussion, the road traffic accident table is constructed by our group and is shown in Table 1. It is inconsistent obviously. The total number of data items is 15; among them only items 1 and 2 are consistent and others are inconsistent.

From Table 1, the partition *π*_*D*_ derived can be written as

(6) D1={x∈U ∣ g(x)=N}={x1,x5,x6,x9};D2={x∈U ∣ g(x)=S}={x2,x3,x4,x7,x8,x10,x11,x12,x13,x14};D3={x∈U ∣ g(x)=L}={x15}.

The partition *π*_*C*_ or equivalent classes IND(*C*) derived by condition attribute set *C* can be written as

(7) IND(C)={{x1},{x2},{x3,x4,x5},  {x6,x7,x8},{x9,x10,…,x13},{x14,x15}}.

The positive and boundary regions of decision attribute with respect to condition attribute set *C* are as follows.

Positive region is

(8) $$\begin{array}{c} {\text{POS}}_{\pi_{C}}\left(\pi_{D}\right)=\left\{\left\{x_{1}\right\},\left\{x_{2}\right\}\right\}. \end{array}$$

Boundary region is

(9) BNDπC(πD)={{x3,x4,x5},{x6,x7,x8}, {x9,x10,…,x13},{x14,x15}}.

The quality of classification or the degree of dependency of *π*_*D*_ on *π*_*C*_ defined by Pawlak in Table 1 is as follows: *γ*_*π*_*C*__(*π*_*D*_) = 2/15.

The *π*_*C*_-roughness of classification is given below. Its value denotes the uncertain percentage of the discourse.

*π*_*C*_-roughness is

(10) $$\begin{array}{c} \rho_{\pi_{C}}\left(\pi_{D}\right)=\frac{\left|{\text{BND}}_{\pi_{C}}\left(\pi_{D}\right)\right|}{\left|U\right|}=\frac{13}{15}=0.867. \end{array}$$

The quality of classification, *γ*_*π*_*C*__(*π*_*D*_), denotes the percentage of objects to all objects in universe that are certainly classified to a decision class. We can use *ρ*_*π*_*C*__(*π*_*D*_), namely,1 − *γ*_*π*_*C*__(*π*_*D*_), to denote the percentage of uncertainly classified objects to all objects in universe and call it the quality of classification uncertainty. These two parameters of inconsistent decision table retain constant in reductions which preserve the positive region invariant.

The rules generated from Table 1 are listed in Table 2. These are primitive rules obtained from original data. The rule has the form *P* → *κQ*, denoting that the confidence of the classification rule is *κ* on the value of attributes set *P*. The first rule in line one from the top can be written as (*a* = *N*, *b* = *N*, *c* = *N*, *d* = *G*, *e* = *G*, *f* = *N*) → _1_ *g* = *N*.

From all 15 data items, 10 rules are achieved as shown in Table 2. The first two of them are certainty rules, and other 8 possible rules are uncertainty rules and appear in four pairs. The four pairs are rules 3 and 4, rules 5 and 6, rules 7 and 8, and rules 9 and 10. The confidence degree of each rule is also listed in the table.

## 3. Uncertainty Analysis of Relative Reductions

There are various definitions of relative reductions in rough set theory [2–8, 11, 12]. All of them can be classified into three categories: region preservation, information preservation, and partition preservation.

Five relative reductions [2–6] chosen from the three categories will be investigated for uncertainty analysis in the following four aspects (Table 3). They are classical positive region reduction proposed by Pawlak [2], mutual information preservation reduction proposed by Miao [4], distribution reduction proposed by Slezak [5], general decision reduction proposed by Kryszkiewicz [6], and boundary partition reduction proposed by Miao et al. [3]. Related articles may be inquired for better explanation of these relative reductions.

### 3.1. The Relationships among Relative Reducts

A reduct is a minimum subset of attributes that provides the same description or classification ability as the entire set of attributes. The relationship among different relative reducts has been studied by many researchers. In order to describe the relationship among the five properties preservation reductions, some definition is given in the first place.

**Definition 7 .**

Given a decision table *S* = {*U*, *A* = *C* ∪ *D*, {*V*_*a*_∣*a* ∈ *A*}, {*I*_*a*_∣*a* ∈ *A*}}, its property *ψ*_1_ preservation reduct is *R*_1_ and property *ψ*_2_ preservation reduct is *R*_2_.

1. If *R*_2_ always preserves property *ψ*_1_ simultaneously, then *R*_2_ is defined as a *ψ*_1_ reduct container.
2. If *R*_2_ is a *ψ*_1_ reduct container, then *ψ*_2_ reduction is defined as a stronger one and *ψ*_1_ reduction is a weaker one.

Because the reduct of propertypreservation is nonexclusive, we cannot say that the reduct of a weaker reduction is included in the reduct of a stronger one. We can only say that if *ψ*_2_ reduction is stronger than *ψ*_1_ reduction, then ∃ reducts *R*_2_ and *R*_1_ of properties *ψ*_2_ and *ψ*_1_ reduction, and *R*_1_⊆*R*_2_.

According to Definition 7, some research results can be assembled here as the following theorems in inconsistent decision tables. The proof of these theorems can be seen in listing reference documents.

**Theorem 8 .**

Given a decision table *S* = {*U*, *A* = *C* ∪ *D*, {*V*_*a*_∣*a* ∈ *A*}, {*I*_*a*_∣*a* ∈ *A*}}, the boundary partition preserving reduct must be a distribution preserving reduct container [11].

**Theorem 9 .**

Given a decision table *S* = {*U*, *A* = *C* ∪ *D*, {*V*_*a*_∣*a* ∈ *A*}, {*I*_*a*_∣*a* ∈ *A*}} and any subset *B* of attributes *C*, *B*⊆*C*, then *H*(*B*∣*D*) = *H*(*C*∣*D*) if and only if ∀*x* ∈ *U*, ∃*τ*_*B*_(*x*) = *τ*_*C*_(*x*)  [13].

This means that a distribution reservation reduction is equivalent to mutual information preserving reduction. The distribution reservation reduction will be used in later of this paper as these two reductions.

**Theorem 10 .**

Given a decision table *S* = {*U*, *A* = *C* ∪ *D*, {*V*_*a*_∣*a* ∈ *A*}, {*I*_*a*_∣*a* ∈ *A*}}, the distribution preserving reduct must be a general decision preserving reduct container [12].

**Theorem 11 .**

Given a decision table *S* = {*U*, *A* = *C* ∪ *D*, {*V*_*a*_∣*a* ∈ *A*}, {*I*_*a*_∣*a* ∈ *A*}}, a general decision preserving reduct must be a positive preserving reduct; the boundary partition preserving reduct must be a general decision preserving reduct container [3].

**Figure 1:**
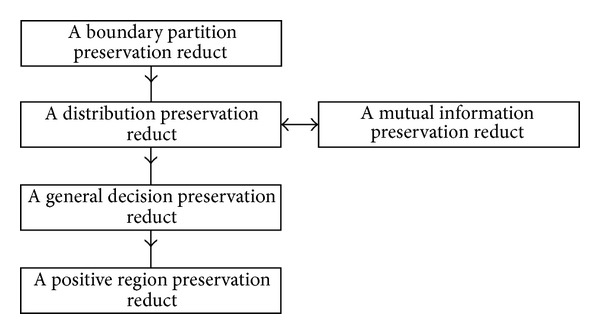
Relations of five relative reducts.

For good understanding, the relationships among five relative reductions are stated in Figure 1 in decision tables. The two-headed arrow expresses the equivalent relation and one-way arrow expresses the container relation. As shown in the figure, a distribution reservation reduct is equivalent to mutual information preserving reduct. A boundary partition reservation reduct must be container for any other four reducts. In other words, in the five relative reductions, a boundary partition reservation reduction is the strongest; a positive region reduction is the weakest.

Five reducts of decision (Table 1) can be computed according to their reduction definitions. The computed results are as follows.

(i) A positive region reservation reduct is
  (11) $$\begin{array}{c} {\text{RED}}_{\text{POS}}=\left\{a,b\right\}. \end{array}$$
(ii) A general decision reservation reduct is
  (12) $$\begin{array}{c} {\text{RED}}_{\text{GEN}}=\left\{a,b,c\right\}. \end{array}$$
(iii) A distribution or a mutual information reservation reduct is
  (13) $$\begin{array}{c} {\text{RED}}_{\text{DIS}}={\text{RED}}_{\text{MIF}}=\left\{a,b,c,d\right\}. \end{array}$$
(iv) A boundary partition reservation reduct and boundary region are
  (14) $$\begin{array}{c} {\text{RED}}_{\text{BPA}}=\left\{a,b,c,d,e\right\}. \end{array}$$

In the process of computing the above five reducts, the attribute adding guideline is adopted. So the reducts meet set inclusion relationship as the reduction becomes stronger. That is,

(15) $$\begin{array}{c} \text{RE}{\text{D}}_{\text{POS}}\subseteq \text{RE}{\text{D}}_{\text{GEN}}\subseteq \text{RE}{\text{D}}_{\text{DIS}}\subseteq \text{RE}{\text{D}}_{\text{BPA}}. \end{array}$$

This expression is not always correct because of the nonexclusive reduct of a reduction.

### 3.2. The Boundary Areas of Five Reductions

Now, the problem will be investigated further from another view. From Theorem 3, we know that the universe of discourse is the sum of positive region and boundary region. Because all the five reductions discussed in this paper preserve the same positive region with the original table (see Section  3.1), the boundary regions of the five reductions are also the same from the view of object set. Now we examine the granularity in the boundary regions from granular computing. The computation results are as follows.

(i) The reduct and boundary region of positive region reduction are
  (16) $$\begin{array}{c} {\text{RED}}_{\text{POS}}=\left\{a,b\right\},\;\;{\text{BND}}_{\text{POS}}=\left\{x_{3},x_{4},\ldots ,x_{15}\right\}. \end{array}$$
(ii) The reduct and boundary region of general decision reduction are
  (17) $$\begin{array}{c} {\text{RED}}_{\text{GEN}}=\left\{a,b,c\right\},\\ {\text{BND}}_{\text{GEN}}=\left\{\left\{x_{3},x_{4},\ldots ,x_{13}\right\},\left\{x_{14},x_{15}\right\}\right\}. \end{array}$$
(iii) The reduct and boundary region of distribution reduction or mutual information reduction are
  (18) REDDIS={a,b,c,d},BNDDIS={{x3,x4,…,x8},{x9,x10,…,x13},{x14,x15}}.
(iv) The reduct and boundary region of boundary partition reduction are
  (19) REDBPA={a,b,c,d,e},BNDBPA={{x3,x4,x5},{x6,x7,x8},{x9,x10,…,x13},{x14,x15}}.

**Figure 2:**
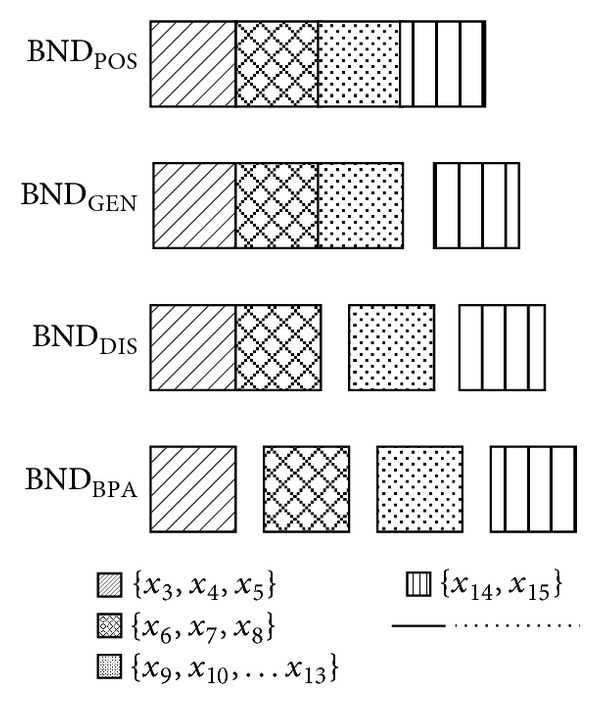
Boundary regions of five relative reductions.

The boundary regions of five relative reductions are illustrated in Figure 2. By observing the granularity of the boundary regions, we can get the following conclusion.

**Proposition 12 .**

If *ψ*_1_ reduction is weaker than *ψ*_2_ reduction and *R*_1_⊆*R*_2_, then the knowledge on *ψ*_2_ reduction is finer than the knowledge on *ψ*_1_ reduction or the knowledge on *ψ*_1_ reduction is coarser than the knowledge on *ψ*_2_ reduction.

In RST, if *R*_1_ and *R*_2_ are two attribute sets, and *R*_1_⊆*R*_2_, then we have IND(*R*_1_)⊇IND(*R*_2_). So Proposition 12 can directly result from Pawlak's definition of knowledge granule in reference [18].

### 3.3. The Classification Rules in Five Relative Reductions

The quality of classification of rules may be measured by rule confidence. Confidence is used to estimate the degree of validity of rules. It means the fraction of objects satisfying both the premises and the conclusions of rules in the set of all objects satisfying the premises of rules. In one rule pair, the higher the confidence of a rule is, the more valid the rule is.

All rules generated from five relative reducts and their confidences are shown in Table 4. This table is very complicated because so much information assembled in it. The upper section of the table contains all rules with premises (shown as Cond. attr.), conclusions (shown as Dec. attr.), and confidence. The lower section of the table expresses the inclusion relationship of upper cells and a specific reduction.

Referring to the fourth row from the bottom, condition attributes aligning with cell, Pos reduct, are the reduct of positive region reduction, RED_POS_ = {*a*, *b*}. The confidences of five rules generated from positive reduct are listed in the nonzero cells in fourth column from right. Two of them are certain rules. Other three of them are possible rules and appear in one pair which has the same condition attributes but different decision attributes. They can be written in the form:

(20) (a=N, b=N)⟶g1=N,(a=Y, b=N)  ⟶g1=S,(a=Y, b=Y)  ⟶g3/13=N,(a=Y,   b=Y)⟶g9/13=S,(a=Y, b=Y)⟶g1/13=L.

Similarly, condition attributes aligning with cell, boundary partition reduct, in the first row from the bottom are the reduct of boundary partition reduction, RED_BPA_ = {*a*, *b*, *c*, *d*, *e*}. The confidences of ten rules generated from boundary partition reduct are listed in the first column in cell confidence from right. They are the same in second and third rows from the bottom.

The change of uncertainty rules in different relative reductions follows a regular pattern. The pair numbers of possible rules are equal to the reduct attributes value numbers of inconsistent data in the table. The number of rules in one pair equals the number of decision values with the same reduct attributes value.

Uncertainty rules that resulted from different reductions can be compared under certain conditions. Because the reduct in rough sets is nonexclusive, only those uncertainty rules that resulted from reducts with set inclusion relation can be compared. This condition is satisfied for rules as shown in Table 4.

Considering the rules from two different reductions, pairs of uncertainty rules in stronger reduction may be integrated into pairs of uncertainty rules in weaker one. In other words, the pair number of uncertainty rule in stronger reduction is larger than or equal to that in weaker one. The number of rules in one pair remains unchanged or gets larger in weaker reduction because of more decision values.

The rule confidence of new formed pair changes because some rules are combined into new pairs and the data item supporting the rules combined at the same time. As an example, when changing from GDR reduction to POS reduction, two pairs of possible rules become one pair. In combination with rule pairs of confidence (3/11, 8/11) and (1/2, 1/2), the reduct value numbers decrease from two to one and the decision values increase from two to three. Combining the data items of previous two pairs with the same decision value, new rules confidences are calculated. They are 9/13 = (8 + 1)/(11 + 2), 3/13 = (3 + 0)/(11 + 2), and 1/13 = (1 + 0)/(11 + 2) as shown in Table 4.

The rule confidence only makes sense in its pairs. Let us inspect the last row in the upper part; rule confidence based on the positive preservation is 1/13 and the general decision preservation is 1/2. It never means that the later rule is more reliable than the former. Rule confidence denotes that if an object meets the preconditions, then it has the probability to have the conclusion. The rule confidence in different pairs cannot be compared.

### 3.4. The Uncertainty Measure of Rough Sets

The uncertainty measure is an important subject in RST. Various definitions of uncertainty measure have been proposed by many researchers [16, 18–21]. All these measures can reflect the uncertainty of relative reductions to some extent.

Upon the rough entropy definition of Theresa B in 1998 and *π*_*B*_-roughness of classification/approximation in Section 2, we give the rough entropy Definition 13 for classification task. Both the roughness and the granularity are concerned in this measure.

**Definition 13 .**

Given a decision table *S* = {*U*, *A* = *C* ∪ *D*, {*V*_*a*_∣*a* ∈ *A*}, {*I*_*a*_∣*a* ∈ *A*}}, a condition attribute set *B*⊆*C*, the rough entropy *Er*_*π*_*B*__(*π*_*D*_) of a classification is calculated by

(21) $$\begin{array}{c} Er_{\pi_{B}}\left(\pi_{D}\right)=\left(\rho_{\pi_{B}}\left(\pi_{D}\right)\right)\frac{\sum_{j_{D_{j}\in U/D}}^{}\sum_{i}^{}\left(Q_{ij}\log\left(P_{i}\right)\right)}{\left|U/D\right|\log\left(1/\left|U\right|\right)}. \end{array}$$

The term *ρ*_*π*_*B*__(*π*_*D*_) denotes the roughness of the classification of *π*_*D*_ with respect to partition *π*_*B*_ governed by attribute set *B*. The second term is a fraction whose nominator is the summation of rough entropy of each decision class *D*_*j*_, *j* = 1, 2,…, |*U*/*D*|, and denominator is normalized factor to ensure the value of fraction to be less than 1. So the rough entropy is less than the roughness of a classification.

On the basis of road traffic accident table (Table 1) and the reducts of five relative reductions, the computation results are as follows.

From Table 1, the partition *π*_*D*_ derived can be written as

(22) D1={x∈U ∣ g(x)=N}  =  {x1,x5,x6,x9};D2={x∈U ∣ g(x)=S}  ={x2,x3,x4,x7,x8,x10,x11,x12,x13,x14};D3={x∈U ∣ g(x)=L}=  {x15}.

The partition *π*_*C*_ or equivalent classes IND(*C*) derived by condition attribute set *C* can be written as

(23) IND(C)={{x1},{x2},{x3,x4,x5},{x6,x7,x8}, {x9,x10,…  ,x13},{x14,x15}}.

The partition or equivalent classes derived by reducts of five relative reductions are as follows:

(24) IND(RESPOS)={{x1},{x2},{x3,x4,…,x15}},IND(RESGEN)={{x1},{x2},{x3,x4,…,x13},{x14,x15}},IND(RESDIS)={{x1},{x2},{x3,x4,…,x8}, {x9,x10,…,x13},{x14,x15}},IND(RESBPA)={{x1},{x2},{x3,x4,x5},{x6,x7,x8}, {x9,x10,…,x13},{x14,x15}}.

In this example, IND(RES_BPA_) is equal to IND(*C*), so the rough entropy of boundary partition preservation reduction equals that of original data in Table 1. It is just a special case. If two reductions are equivalent, as for distribution preservation reduction and mutual information preservation reduction, all measures for them will be equal.

The values of rough entropy of five relative reductions are worked out and shown in Table 5. The rough entropy  *Er*_*π*_*B*__(*π*_*D*_) of a classification in Definition 13 can be used effectively to describe the uncertainty measure. The results meet Proposition 14 very well.

**Proposition 14 .**

In an inconsistent decision table, the uncertainty of classification will increase after a reduction. The weaker is the reduction; the lager is its rough entropy (uncertainty of classification).

When a problem is resolved at a reduced attributes set after a reduction, its computational complexity decreases and uncertainty increases. This uncertainty variance can be observed from the following four aspects. The first one is the reduct, a reduced attribute set. The second one is the granularity of the universe of discourse, including positive and boundary regions. The third one is the number of rule pairs. The last is reflected in the measure, rough entropy of a classification.

## 4. Conclusion

A relative reduct is a minimum set of attributes that keeps a particular classification property. Different property preservation reductions bring about different reducts. In this paper, the uncertainty analysis of five relative reductions in an inconsistent decision table is discussed for classification tasks from four aspects. In the former three points, the relationships among reduct sets, boundary regions, and rules produced by five relative reductions are compared, respectively. In the last section rough entropy, a measure of uncertainty for a decision table is proposed for classification task. The conclusion is obtained that the weaker reduction has more uncertainty than the stronger one in an inconsistent decision table.

## Figures and Tables

**Table 1:** Road traffic accident table.

Instant	Condition attributes						Decision attribute
	a	b	c	d	e	f	g
1	N	N	N	G	G	N	N
2	Y	N	N	G	G	N	S
3	Y	Y	N	G	G	N	S
4	Y	Y	N	G	G	N	S
5	Y	Y	N	G	G	N	N
6	Y	Y	N	G	B	Y	N
7	Y	Y	N	G	B	Y	S
8	Y	Y	N	G	B	Y	S
9	Y	Y	N	B	B	Y	N
10	Y	Y	N	B	B	Y	S
11	Y	Y	N	B	B	Y	S
12	Y	Y	N	B	B	Y	S
13	Y	Y	N	B	B	Y	S
14	Y	Y	Y	B	B	Y	S
15	Y	Y	Y	B	B	Y	L

Notes: ① a: drunk, b: tired, c: speedy, d: weather, e: road, f: mountains, and g: accident.

② Y: yes, N: no, G: good, B: bad, S: small, and L: large.

**Table 2:** The confidence of rules in Table 1.

Rules	Conditional attributes						Decision attribute	Validity
	a	b	c	d	e	f	g	*κ*
1	N	N	N	G	G	N	N	1
2	Y	N	N	G	G	N	S	1
3	Y	Y	N	G	G	N	N	1/3
4	Y	Y	N	G	G	N	S	2/3
5	Y	Y	N	G	B	Y	N	1/3
6	Y	Y	N	G	B	Y	S	2/3
7	Y	Y	N	B	B	Y	N	1/5
8	Y	Y	N	B	B	Y	S	4/5
9	Y	Y	Y	B	B	Y	S	1/2
10	Y	Y	Y	B	B	Y	L	1/2

Notes: *κ*-confidence, others are the same as in Table 1.

**Table 3:** Five different reduct objections.

Year	Authors	Reduction objection	Reduction function
1982	Pawlak [2]	Positive region	POS_*B*_(*D*) = POS_*C*_(*D*)
1997	Miao [4]	Mutual information	*I*(*B* : *D*) = *I*(*C* : *D*)
2000	Slezak [5]	Distribution	*μ*_*B*_ *(x)* = *μ*_*C*_ *(x) *
2001	Kryszkiewicz [6]	General decision	*τ*_*B*_(*x*) = *τ*_*C*_(*x*)
2009	Miao et al. [3]	Boundary Partition	IND(*B*/*D*) = IND(*C*/*D*)

**Table 4:** The rules description of five reductions.

Cond. attr.					Dec. attr.	Confidence			
a	b	c	d	e	g	POS	GDR	DR	BPR
N	N	N	G	G	N	1	1	1	1
Y	N	N	G	G	S	1	1	1	1
Y	Y	N	G	G	N	3/13	3/11	2/6	1/3
					S	9/13	8/11	4/6	2/3
		Y		B	S				1/3
					L				2/3
		N	B	B	N			1/5	1/5
					S			4/5	4/5
		Y		B	S		1/2	1/2	1/2
					L	1/13	1/2	1/2	1/2
Pos reduct		5 rules and one pair of R.unc.							
General decision			6 rules and two pairs of R.unc.						
Distribution reduct				8 rules and three pairs of R.unc.					
Boundary partition reduct					10 rules and four pairs of R.unc.				

Notes: Cond. attr., Dec. attr., and R.unc. are abbreviations for condition attributes, decision attributes, and uncertain rules.

**Table 5:** The uncertainty measure of five relative reductions.

Reduction	Raw data	POS	GEN	DIS	BPA
Rough entropy	0.214	0.712	0.395	0.287	0.214
